# Conservative management of voiding dysfunction

**DOI:** 10.4103/0970-1591.32068

**Published:** 2007

**Authors:** Anita Patel

**Affiliations:** Endoskopik Klinik and Hospital, Ameya, Pandurang Naik Rd, Shivaji Park, Mumbai, India

**Keywords:** Incontinence

## Abstract

This review article discusses the efficacy of various conservative therapies in the management of voiding dysfunction with special reference to urinary incontinence. The article emphasizes the fact that conservative therapies have limited side effects and they do not jeopardize future treatment options. Behaviour therapy, pelvic floor therapy and biofeedback; electrical and magnetic stimulation are discussed here individually. Though there is unanimous agreement that these therapies improve quality of life, complete cure is rare. All therapies work better in conjunction with each other rather than in isolation. The review also highlights the need for randomized controlled trials of better methodology.

## PREVALENCE OF URINARY INCONTINENCE

Often described as a social cancer, urinary incontinence and allied voiding dysfunction continue to show rising prevalence. The main reasons for this are increased awareness and diagnosis as well as the growth of the ageing population. Though the statistics in India are not known, 13 million citizens are believed to have urinary incontinence in the US.[1] The overall prevalence of urinary incontinence in 1460 community-dwelling women in the Netherlands was reported as 46%,[2] out of which 12% were classified as having severe incontinence. Affecting every facet of human life, studies have shown a clear link between urinary incontinence and depression.[3] Among different types of incontinence, stress urinary incontinence (SUI) is believed to be the most common with an estimated prevalence of 8-33%.[4] However, urge urinary incontinence (UUI) being unpredictable, affects the quality of life to a greater extent.

## RATIONALE FOR CONSERVATIVE MANAGEMENT

As urinary incontinence is not a life-threatening condition, quality of life takes precedence over other issues when deciding the therapy. According to the “Clinical Guideline Panel” of the US Department of Health and Human services, “surgery, except in very specific cases, should be considered only after behavioral and pharmacological interventions have been tried”.[4] Various conservative options such as behavioral and physical therapy report limited side effects and do not jeopardize future treatment options. Theoretically, physical therapy may also increase the patient's understanding of lower urinary tract function and dysfunction.

This article reviews various conservative treatment options for the management of voiding dysfunction and urinary incontinence. Drug therapy and advanced neuromodulation techniques are not covered in this review.

## TYPES OF CONSERVATIVE THERAPIES

Various conservative therapies rely heavily on the motivation of both the patient and the treating physician / nursing staff. Before starting any therapy, a detailed clinical, urological, and in select situations, neurological examination is recommended. However, invasive testing is rarely required before initiating these conservative treatments.[5] All conservative therapies primarily revolve around educating the patient about the normal and the abnormal with instructions regarding lifestyle modifications, fluid and dietary management, timed voiding, pelvic muscle training and urge inhibition.[6]

### Behavioural Therapy

Behavioral therapies are based on educating the patients to modify their behavior so as to reduce or sometimes even cure the incontinence. Patients suffering from urgency and urge incontinence seem particularly suitable for this form of therapy. The regime starts with maintaining a voiding diary. Very often this reveals excess water drinking as the cause of frequent urination, especially in India. The diary helps in setting the goals of treatment, i.e., decreasing the frequency, prolonging the intervals between urination and avoiding incontinence.

Burgio *et al.* describe detailed techniques of behavioral therapy including timed voiding and techniques to stop / delay urination by sitting or standing quietly and repeatedly contracting the pelvic muscles. Distraction techniques such as deep breathing or mathematical problem-solving were also shown to be helpful.[7] Same authors reported better results with a combination of behavioral therapy and biofeedback in comparison with anticholinergic medication alone. 80.7% patients reported reduced incontinence with behavioural treatment alone as against 68.5% with anticholinergics and 39.4% with placebo. However, it must be noted that both mixed incontinence and SUI patients were included, which explains the better response to behavioral treatment.[8]

Fantl and colleagues studied the potential benefits of a timed voiding schedule in elderly patients with urinary symptoms. In a study of 131 community-dwelling women between the ages of 55 and 90, incontinence episodes were cut in half in 75% of the women following six weeks of intensive education and observation. Reinforcement was given about maintaining a regimented voiding schedule and by progressively prolonging the interval between voiding. Women with both SUI and UUI, either isolated or together, were enrolled in the study and both groups benefited from treatment.[9]

Burgio and colleagues studied three prospective randomized trials of behavioral treatment for incontinence to assess if any factors could predict the outcome of such therapies. A total of 19 potential predictors of outcome were selected for analysis. They concluded that three factors were significantly related to achieving total continence: 1) self-reported frequency of incontinent episodes (*P* = 0.035), 2) frequency of incontinent episodes as measured in the baseline bladder diary (*P*<0.001) and 3) wearing protection (*P*=0.011). Women who reported ten or more incontinent episodes per week in their bladder diaries were much less likely to be completely continent after treatment than were women with fewer than ten episodes per week. Also, those using protection were more likely to have severe incontinence, thus explaining the poorer outcome.[10]

A Cochrane review of behavioral training in UUI [2003 by Wallace *et al*] included a study of 28 reports of ten trials with a total of 1366, predominantly female, participants. Not all participants in five out of ten trials who had overactive bladders, had urinary incontinence. Data from the other five trials with 467 participants, all female, was therefore included in the review. The issues assessed were: Is bladder training better than no bladder training? Is bladder training better than other treatments? Is combining bladder training with another treatment better than that other treatment alone?

They concluded that bladder training may be helpful for the treatment of urinary incontinence, but also added that this conclusion can only be tentative as the trials were of variable quality and of small size. There was also not enough evidence to determine whether bladder training was useful as a supplement to another therapy. The conclusion ended with a remark that “more research is required.”[11]

### Pelvic floor therapy and biofeedback

Pelvic floor muscle (PFM) education is a well-accepted therapy for the treatment of SUI. However, as in behavioral therapy, prerequisites for a successful outcome are motivated patients and therapists. There are two proposed theories for explaining the effectiveness of PFM education for SUI. 1) Women learn to precontract the PFM during any physical stress known to produce SUI, 2) strength training improves muscle volume, which in turn, offers better support.[12] Even in overactive bladder-related incontinence, PFM training (PFMT) is at least theoretically valuable. Firstly, simple sphincteric closure opposes a bladder contraction and minimizes leak. Secondly, local inhibitory reflexes between bladder and pelvic floor musculature are activated. Finally, increased muscle tone and strength should decrease overactivity.[5] Arnold Kegel was the first to describe in detail the methods of pelvic floor rehabilitation and their applications.[13,14] He emphasized the need to ensure that the correct muscles are exercised. In his study, up to 30% of women could not contract these muscles voluntarily. He implemented an intensive supervised program of progressive pelvic floor muscle contractions under supervision that incorporated biofeedback using a perineometer.

Payne proposed an algorithm for individualizing therapy based on simple PFM assessment during clinical examination.[5] Patients are classified into three groups: **1.** Those with minimal or no ability to isolate and contract the PFM, **2.** those who can isolate the muscles with poor strength and **3.** those with good muscle strength and isolation on initial examination. The first group usually needs some form of biofeedback to get started. The second group usually benefits with PFMT with no added advantage with biofeedback. In the last group, the value of PFMT is unclear as their incontinence despite their good musculature, may indicate severe urethral incompetence or bladder dysfunction.

Elser *et al*. studied urodynamic parameters pre- and postbladder training, PFMT and a combination of both types of training. After a 12 week period, they found no difference in urethral pressure profiles or in cystometry. They concluded that the mechanism by which clinical improvement occurs remains unknown.[14]

Cammu *et al.* evaluated 447 women with SUI. All women received supervised PFM training. 22 different patient characteristics were considered for outcome measurement. 49% of women considered the treatment to be successful. Among various parameters, more than 2 leakages per day before treatment and chronic psychotropic medication usage where found to be predictors of failure. The odds for an individual patient to be treated successfully were only 15% when these predictors were present. On the other hand, patients who did not use protective undergarments (67% success) or who were not incontinent on a daily basis (63% success) or who did not leak at the first cough (60% success), predictably had a favorable response to PFMT.[15]

Cammu *et al.* did a postal questionnaire survey of 45 women (mean age 61 yrs) who had received PFMT ten yrs earlier. In 24 (53%) patients, the treatment was successful initially and 16/24 reported satisfactory continence even ten yrs later. Out of 21 (47%) women in whom the initial treatment had failed, 5 women claimed to be much better and 13 had undergone surgery. Thus, 21 women (46%) were happy with the outcome of PFMT 10 years later. Those with long-term improvement in SUI had practiced PFM exercises more regularly and often preemptively just before a physical stress. However, it must be noted that this was a questionnaire survey based on patient's own perception of her incontinence.[17] How else would any study or survey even begin to monitor and assess incontinence in conscious patients?

Berghman *et al.* conducted a systematic review of randomized, controlled trials (RCTs) of conservative treatment of urge incontinence in women. Out of 15 RCTs, eight were considered to be adequate in terms of methodological quality. Based on levels of evidence criteria, the authors reached a conclusion that “although almost all studies reported a favorable effect of physical therapy, more research of high methodological quality is required”. The major problem areas were lack of consistency in the criteria for patient evaluation and the methodology used. They also emphasized that the trials may be biased if the assessor is a healthcare provider himself.[18]

A Cochrane systematic review of PFMT for incontinence studied 13 trials involving 714 women (375 PFMT, 339 controls) but only six out of the 13 trials (403 women) contributed data to the actual analysis. There was considerable variation in interventions used, study populations and outcome measures. The authors concluded that “overall, the review provides some support for the widespread recommendation that PFMT be included in first-line conservative management programmes for women with stress, urge or mixed, urinary incontinence. Statistical heterogeneity reflecting variation in incontinence type, training and outcome measurement made interpretation difficult. The treatment effect might be greater in younger women (in their 40's and 50's) with SUI alone, who participate in a supervised PFMT programme for at least three months, but these and other uncertainties require testing in further trials”.[19]

### Biofeedback

Biofeedback involves the monitoring of a learned or naturally occurring physiological process in a way that information about the process can be given back to the patient. In patients with urinary incontinence and an overactive bladder, biofeedback couples the physiological recruitment of the proper muscle groups in pelvic floor exercises with observable stimuli, typically visual or audio cues. In most cases, either vaginal, perineal or rectal electromyographic monitoring can be used to detect muscle activity, which is reported back to the patient, making them aware of the success of the exercise. Thus biofeedback makes learning PFM exercises easy. In cases of UUI and overactive bladders, patients are instructed to perform a pelvic floor exercise through biofeedback at the time of an urgency episode. In patients with SUI, patients simply learn the PFM exercises through biofeedback and perform them at regular intervals. From a practical standpoint, pelvic floor exercises, biofeedback therapies and behavioral modification techniques, as described earlier, are believed to be complementary to each other.

Vaginal cones are the cheapest form of biofeedback, which can be used for PFMT. The set consists of 5 plastic cones, identical in size and shape but with different weights, from 15 to 85 gms. Patients are taught how to retain the cones inside the vagina, while performing simple activities such as walking, squatting, running, coughing etc, over a 10 minute period twice a day. The goal eventually is to retain the heaviest cone during these activities and prevent it from falling out.

AS systematic review by Herbison *et al.* studied the effects of weighted vaginal cones in women with SUI. Out of various trials published between 1966 and 1999, only ten trials met the inclusion criteria. The overall outcome of this survey was that the results were better with cones than with the control treatment and the effect achieved was comparable with PFMT alone or with electrical stimulation.[20]

In a systematic review of RCTs of conservative therapy for UUI, Berghman *et al*. concluded that there are too few studies to evaluate effects of PFM exercise with or without biofeedback for women with UUI.[18]

### Electrical stimulation

It is believed that overdistension neuropathy of the PFMs during delivery with consequent Wallerian degeneration may result in severe damage to the nerve supply of PFM and urethral sphincter. Electrical stimulation may improve re-innervation or some type of electrical reorganization in the damaged areas.[21]

Two types of electrical stimulation have been used to treat female SUI: chronic [below sensory threshold stimulation] and high intensity [maximum tolerated stimulation with no pain.] The latter is usually used once or twice daily for 15-20 minutes at higher frequencies [> 20-50 Hz]. Stimulation can be applied with vaginal, anal or surface electrodes. However, the results for cure or improvements vary.[22] In a placebo controlled trial with a sham device, Sand *et al.* showed a 62% improvement in patients actually using electrical stimulation. However, only 20% became dry (cured of incontinence).[23] Rapid magnetic stimulation can also be used to deliver electrical energy to pelvic floor structures. Magnetic stimulation can easily be applied to the pelvic floor using a magnetic coil fitted to the base of a chair. Thus, the therapy becomes noninvasive and hence, appealing to the patient as compared to electrical stimulation. Bradshaw *et al.* studied the acute effect of magnetic stimulation on involuntary detrusor activity during natural; filling. Two natural filling cycles were studied; one unstimulated and the second stimulated using the Neotonus magnetic chair. Though there was statistically significant improvement in cystometric capacity and the amplitude and duration of overreactivity, this did not translate into a clinically consistent change in symptoms.[24]

Chandi *et al.* evaluated magnetic stimulation as a treatment for female urinary incontinence. Both urge and mixed incontinence patients were included in the study. Treatment was given twice a week for eight weeks. Outcome was assessed by pad test, patient satisfaction as well as urodynamics. This revealed objective improvement in 58% of patients. However, the sample size was small (24 patients) with no control group and the follow-up was short.[25]

## SUMMARY

Conservative therapies for urinary incontinence are safe with no documented side effects. Literature reports give conflicting opinions regarding their efficacy. Though there seems to be unanimous agreement that these therapies improve the quality of life, a complete cure is uncommon. Various therapies work better when used in conjunction with each other. Systematic reviews of each aspect of conservative therapy point towards a lack of good quality trials. The consensus in such reviews is that there is a need for RCTs of better methodology.
